# Antimonene with two-orders-of-magnitude improved stability for high-performance cancer theranostics†
†Electronic supplementary information (ESI) available. See DOI: 10.1039/c9sc00324j


**DOI:** 10.1039/c9sc00324j

**Published:** 2019-04-02

**Authors:** Guihong Lu, Chengliang Lv, Weier Bao, Feng Li, Fan Zhang, Lijun Zhang, Shuang Wang, Xiaoyong Gao, Dongxu Zhao, Wei Wei, Hai-yan Xie

**Affiliations:** a School of Life Science , Beijing Institute of Technology , No. 5 South Zhong Guan Cun Street , Beijing 100081 , China . Email: hyanxie@bit.edu.cn ; Email: weiwei@ipe.ac.cn; b State Key Laboratory of Biochemical Engineering , Institute of Process Engineering , Chinese Academy of Sciences , 1 North 2nd Street, Zhong Guan Cun , Beijing 100190 , China

## Abstract

Although the antimonene (AM) nanomaterial is recently emerging as a new photothermal therapy (PTT) agent, its rapid degradation in physiological medium immensely limits its direct utilization. To this end, we herein engineered AM by the cooperation of dimension optimization, size control, and cell membrane (CM) camouflage. Compared with traditional AM nanosheets, the resulting AM nanoparticles (∼55 nm) cloaked with the CM (denoted as CmNPs) exhibited significantly improved stability and increased photothermal efficacy as well as superior tumor targeting capacity. After intravenous injection, the CmNPs enabled satisfactory photoacoustic/photothermal multimodal imaging at tumor sites. Meanwhile, the PTT together with the newly explored function of photodynamic therapy (PDT) achieved a potent combination therapy with few side effects. The maximized theranostic performance thus strongly recommends CmNPs as a safe and highly reliable modality for anticancer therapy.

## Abstract 2

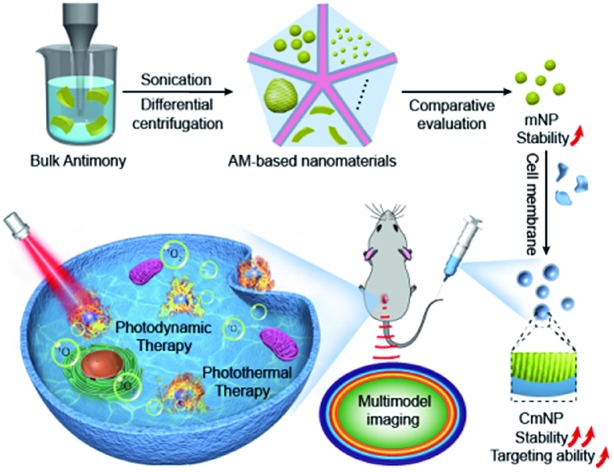
Antimonene was engineered with significantly improved stability, superior tumor targeting capacity and increased photothermal efficacy for high-performance cancer theranostics.

## Introduction

Exploring new materials with special functions has provided efficient ways for cancer diagnosis and treatment,^1^–^4^ such as photoacoustic (PA) imaging,^5^,^6^ magnetic resonance imaging (MRI),^7^,^8^ photothermal therapy (PTT),^9^,^10^ and photodynamic therapy (PDT).^11^,^12^ Recently, antimonene (AM) has emerged as a new candidate for cancer theranostics due to its extremely strong light absorption in the NIR region.^13^–^15^ This property endows it with the capacity of high photothermal conversion efficiency (AM quantum dots: 45.5%; AM nanosheets: 41.8%),^16^,^17^ which was much higher than those of many other nanomaterial-based photothermal agents such as graphene oxide, Au nanorods, Au nanoshells and BP quantum dots. The high PTCE also resulted in sensitive PA signals *in vivo*.^17^ Considering that most photothermal materials show a capacity for ^1^O_2_ generation,^18^,^19^ the possibility of utilizing AM as a PDT agent thus exists, and this feature could further augment the anticancer performance of AM but remains unexploited.

Unfortunately, the direct utilization of AM for cancer theranostics is rare so far. One of the most critical issues is its rapid degradation in physiological medium.^20^,^21^ Such poor stability not only closes the time window to play a therapeutic role in tumors, but also raises safety concerns.^22^,^23^ As AM degradation highly depends on oxygen,^21^ one efficient strategy to overcome this impediment is likely to decrease the specific surface area, which can be realized by regulating the shape and size.^24^,^25^ The other feasible way may be surface modification, which can shield nanomaterials from the medium.^26^–^30^ Due to the lack of reactive groups on the AM substance surface, traditional chemical reaction-based approaches seem impossible. To date, the only reported method for AM modification is coating with DSPE-PEG *via* van der Waals forces and hydrophobic interactions.^16^,^17^ Although effective, this approach has not yet provided the silver bullet for full utilization *in vivo*. For example, the gradual exfoliation of these physically bound DSPE-PEG molecules is inevitable, which undoubtedly compromises the improvement in stability.^31^ Additionally, an unexpected immunogenic response known as “accelerated blood clearance (ABC)” has been observed with PEGylated nanocarriers, resulting in increased clearance during repeated administration.^32^,^33^ In addition, considering the stochastic nature of the enhanced permeability and retention (EPR) effect, an active targeting capacity is further needed to improve AM accumulation at tumor sites.^34^–^36^

**Scheme 1:**
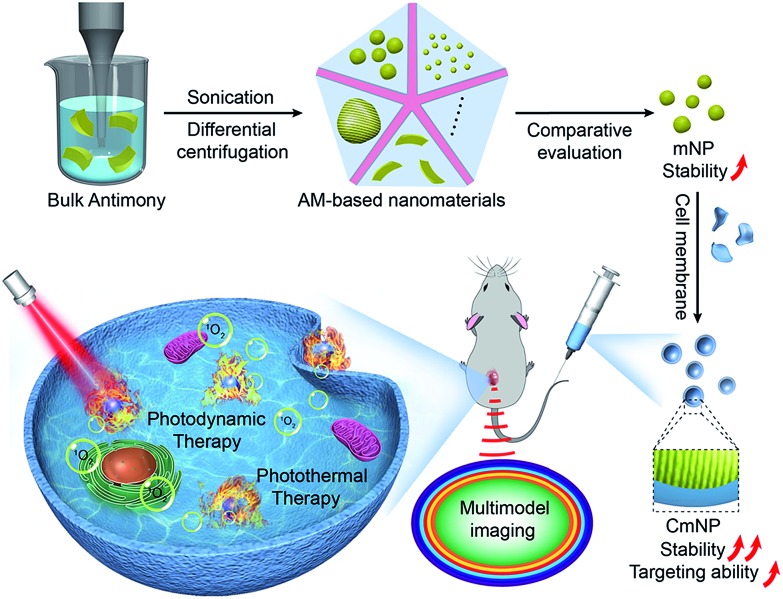
Schematic illustration of the preparation of cancer cell membrane-camouflaged AM nanoparticles (CmNPs) and their application in multimodality imaging-guided combinatorial cancer therapy.

To improve the stability and thus maximize the theranostic performance *in vivo*, we herein engineered AM by using a synergic approach, which included dimension optimization, size control, and surface camouflage (Scheme 1). Typically, distinct ultrasonic probes with high-power liquid exfoliation were utilized to prepare AM nanoparticles (NPs) and nanosheets (NSs), which could be separated with uniform size by differential centrifugation. Upon comparative evaluations, AM NPs with a size of ∼55 nm outperformed other candidates in terms of suitable size to utilize the EPR effect and superior stability for phototherapy. Subsequent camouflaging of the cancer cell membrane (CM) would not only further improve stability but also endow mNPs with good tumor targeting capacity. After intravenous injection, the resulting CmNPs could accumulate at tumor sites due to their homotypic tumor self-recognition ability. Once exposed to NIR light, photoacoustic/photothermal multimodal imaging enabled noninvasive guidance. Additionally, efficient heat and ^1^O_2_ were generated for PTT and PDT, respectively. As a result, complete tumor inhibition could be achieved with few abnormalities, showing great promise for utilizing CmNPs as a new modality to fight against cancer.

## Results and discussion

### Synthesis, characterization and stability comparison of AM NSs and NPs

**Fig. 1:**
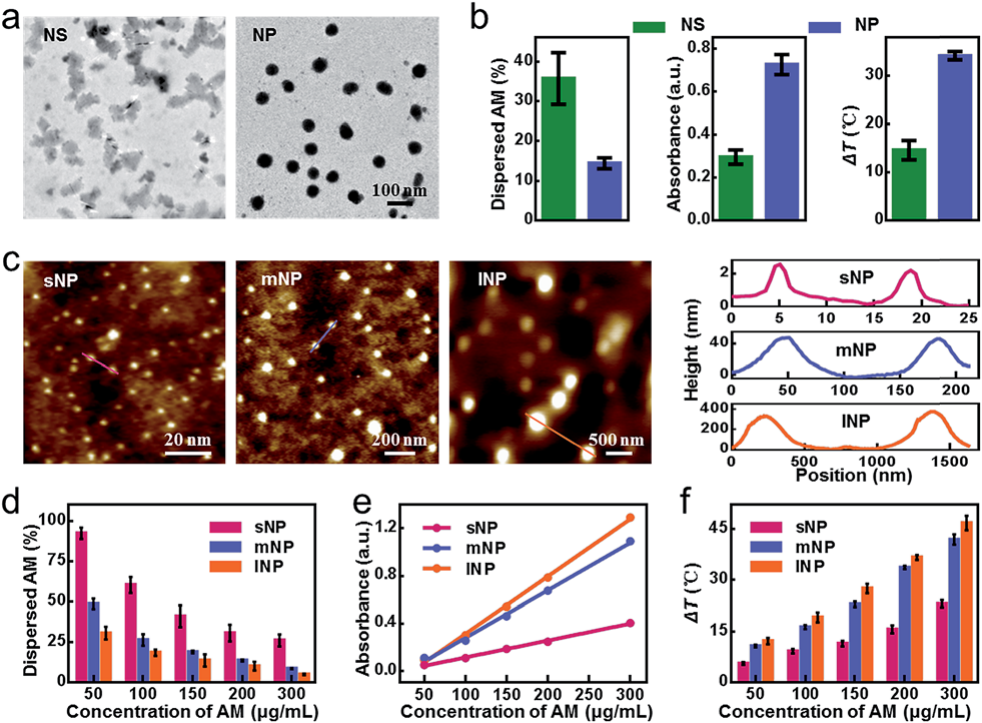
Characterization and stability comparison of AM NSs and NPs. (a) TEM images of NPs and NSs. (b) Stability, absorbance intensity and temperature change of NPs and NSs under the same conditions. (c) AFM images and thickness of NPs with different sizes (the samples were coated onto the mica film before imaging). (d) Stability evaluation of NPs by detecting SbxOy in the solution under ambient conditions. (e and f) Absorbance intensity and temperature changes of different-sized NPs with variable concentrations (808 nm laser, 0.8 W cm^–2^, 5 min). All data represent the means ± s.d. (*n* = 3).

AM NSs can be simply exfoliated from bulk powder through a modified two-step liquid exfoliation strategy, which involves ultrasound probe sonication together with ice-bath sonication in *N*-methyl-2-pyrrolidone. Unfortunately, the high specific surface area sourced from two dimensions (2D) facilitates the exposure of AM NSs to oxygen in the medium, thus accelerating the degradation process. To decrease the specific surface area, we utilized an ultrasound probe with a distinct jagged head and obtained AM NPs. For comparative evaluation, the obtained AM NSs and NPs were further separated with uniform size (∼55 nm, Fig. 1a) by differential centrifugation. Compared with traditional 2D AM NSs, 3D AM NPs halved the dissolution rate (Fig. 1b), indicating their superior stability in medium. Correspondingly, we observed significant increases in the absorbance at 808 nm and temperature after irradiation.

Having demonstrated the benefit of 3D, we next examined the effect of particle size, which was another important factor correlated with the specific surface area of NPs. Taking the aforementioned AM NPs as a middle reference (denoted as mNPs), we also prepared small AM NPs (sNPs, ∼2 nm) and large AM NPs (lNPs, ∼295 nm) by differential centrifugation for further comparative evaluation (Fig. 1c, S1 and S2†). Compared with mNPs, sNPs with a 24-fold increase in specific surface area exhibited a significantly accelerated dissolution rate, while an evidently decreased absorbance at 808 nm and temperature after irradiation was observed (Fig. 1d–f and S3†), which excluded the feasibility of efficient phototherapy. In contrast, the 5-fold decrease in the specific surface area of lNPs resulted in moderately improved stability and photothermal effects. Based on this *in vitro* performance alone, lNPs seemed to be slightly superior to mNPs. However, the oversize of lNPs might, in turn, remarkably compromise the EPR effect due to the limited fenestration space of newly formed tumor vessels.^34^ Taking these aspects together into consideration, mNPs with a suitable size for the EPR effect and good stability for phototherapy thus shed a brighter light on their *in vivo* performance.

### Characterization and stability evaluation of CmNPs

**Fig. 2:**
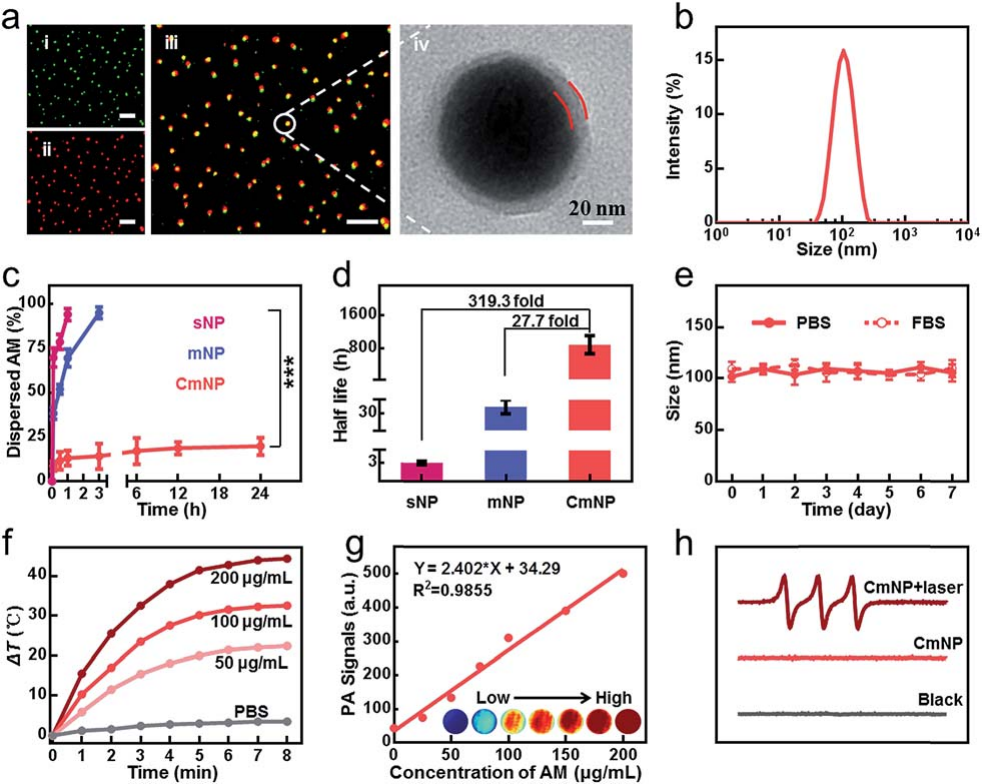
Characterization of CmNPs. (a) CLSM and TEM images of CmNPs (i–iii: green: NPs, red: CM, scale bar: 10 μm). (b) Hydrodynamic diameter of CmNPs. (c) Variation of the dispersed NPs after being stored in PBS for different periods of time (AM: 100 μg mL^–1^). (d) The degradation half-life of NPs. (e) Size stability of CmNPs in PBS or 10% FBS. (f) Time-dependent temperature increase in CmNP solutions with different concentrations during NIR laser irradiation (808 nm laser, 0.8 W cm^–2^). (g) *In vitro* PA images and linear fit of photoacoustic intensity to the concentration of CmNP aqueous solution. (h) ESR spectra of CmNPs under different conditions. All data represent the means ± s.d. (*n* = 3).

Although improved, the stability of mNPs was still unsatisfactory. Even at a high concentration of 300 μg mL^–1^, only 47.9% mNPs remained undispersed in PBS within 24 h, leading to a significantly decreased photothermal efficacy (Fig. S4†). To go a step further, we continued to consider shielding mNPs from the medium by surface modification. Instead of DSPE-PEG adsorption, we herein utilized the cancer cell membrane to physically enwrap mNPs, which not only mitigated the exfoliation during AM dissolution but also provided tumor targeting capacity *via* homotypic tumor self-recognition. As shown in Fig. 2a and S5†, the particle size, surface charge and protein of the CM coated nanoparticles, the colocalization of mNPs/CM signals in the confocal laser scanning microscopy (CLSM) images and the membrane layer around mNPs in the TEM image together demonstrated the successful CM camouflage. Especially, the outer lipid shell thickness (∼8 nm) in the TEM image and the increase in the hydrodynamic diameter (∼20 nm) after CM coating indicated the single layer membrane coating on the surface of AM NPs.^37^,^38^ CmNPs remained well dispersed with an average hydrodynamic diameter of approximately 106 nm (Fig. 2b), which was favorable for intravenous injection and the EPR effect. More importantly, the CmNPs exhibited a much improved particle stability (Fig. S4 and S6†). Taking the concentration of 100 μg mL^–1^ as an example, almost all sNPs and mNPs had dispersed within 1 h and 3 h at 37 °C, respectively (Fig. 2c). In contrast, more than 80% CmNPs remained stable even over 24 h. The degradation half-life of CmNPs increased over 27.7-fold compared with that of mNPs, and this value could jump to 319.3-fold compared with that of sNPs (Fig. 2d). Correspondingly, the particle size, surface zeta potential, and polydispersity index (PDI) of CmNPs showed little change in one-week storage in PBS and 10% FBS medium (Fig. 2e, S7†) if stored at 4 °C. Such superior stability further endowed CmNPs with outstanding photoproperties.

As shown in Fig. 2f and S8,† the photothermal heating curves showed strong concentration- and time-dependent photothermal effects for CmNPs, and the temperature increment (Δ*T*) was up to 44.5 °C at a concentration of 200 μg mL^–1^ after 8 min of irradiation with a low power laser (0.8 W cm^–2^). Accordingly, the high photothermal effect resulted in a strong PA signal that showed a good linear relationship with the concentration (Fig. 2g). Meanwhile, typical 1 : 1 : 1 triplet signals in electron spin resonance (ESR) spectra (Fig. 2h) as well as the decreased signal of ABDA and DPBF (Fig. S9 and S10†) revealed the capacity of efficient ^1^O_2_ production. Moreover, we found that CmNPs had outstanding biocompatibility. They could be rapidly degraded *via* NIR irradiation as usual (Fig. S11†), and more than 90% of the particles could be gradually dispersed in 7 days at 37 °C even without NIR-irradiation (Fig. S12†). As a result, no obvious *in vitro* and *in vivo* toxicity was observed (Fig. S13 and S14†). The aforementioned performance of CmNPs together paved the way for their *in vivo* application.

### Specific targeting capability of CmNPs

**Fig. 3:**
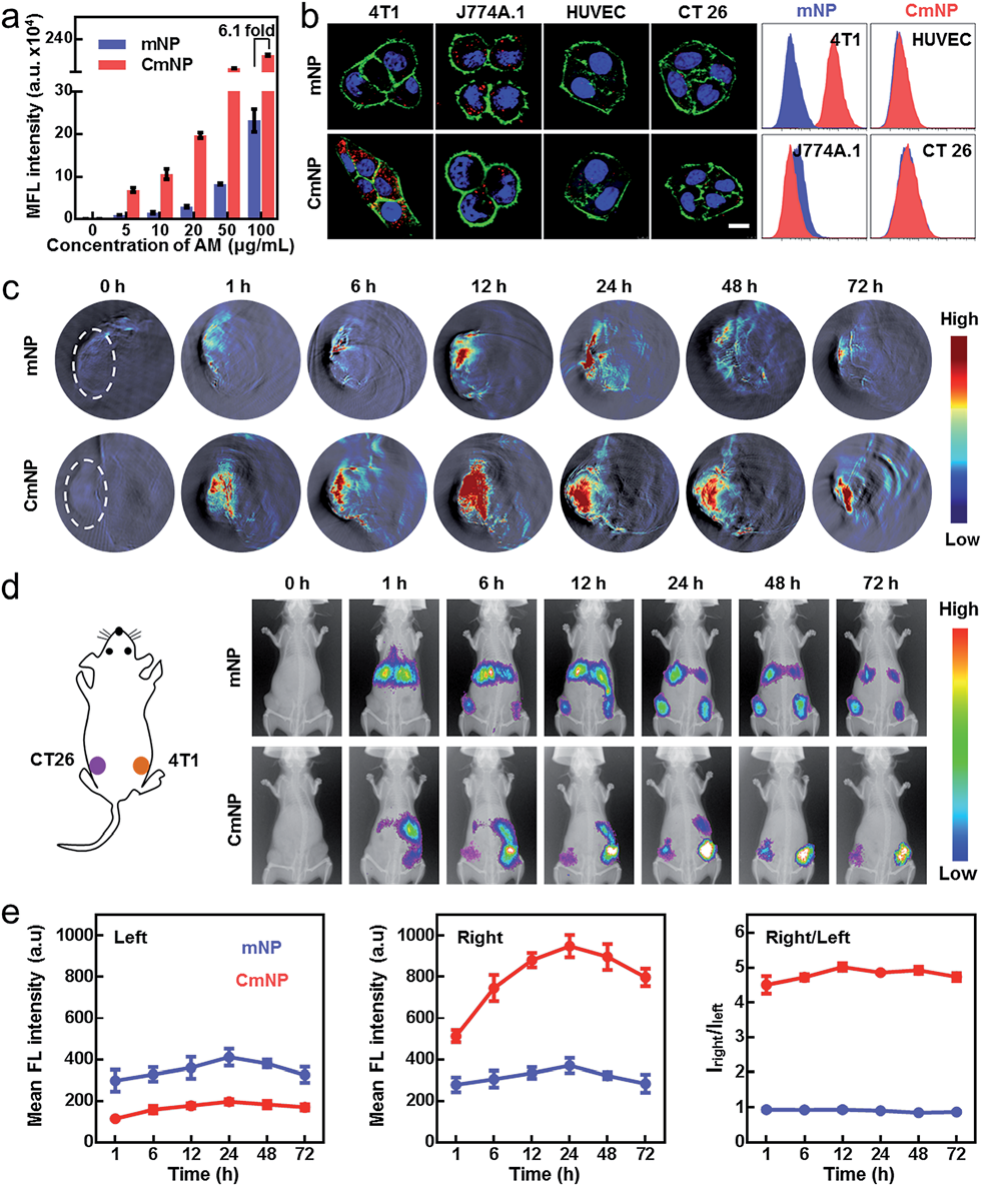
Specific targeting capability of CmNPs. (a) Intracellular uptake of mNPs or CmNPs by 4T1 cells. (b) CLSM images and the corresponding flow cytometry analysis of 4T1, J774A.1, HUVEC and CT26 cells after incubation with mNPs or CmNPs (scale bar: 5 μm. Red: NP, blue: nuclei, green: CM. The concentration of AM in all cases was 20 μg mL^–1^). (c) *In vivo* real-time PA imaging of 4T1 tumor-bearing mice after being injected (i.v.) with mNPs or CmNPs. (d) *In vivo* real-time fluorescence images of Balb/c mice simultaneously bearing 4T1 (right) and CT26 (left) tumor xenografts after being injected (i.v.) with mNPs or CmNPs. (e) Quantitative time dependent distributions of NPs in 4T1 or CT26 tumors and their intensity ratio. All data represent the means ± s.d. (*n* = 3).

In addition to the improved stability, another benefit sourced from the camouflage of the cancer CM could be the tumor targeting capacity. To explore this idea, we next systematically investigated the effect of CM cloaking on the uptake profiles. As shown in Fig. 3a, camouflaging mNPs with the 4T1 breast cancer CM (4T1/CmNPs, also denoted as CmNPs) significantly increased the cellular uptake into 4T1 cells. Taking the dose of 100 μg mL^–1^ as an example, the increment of intracellular AM (detected by ICP) yielded 6.1-fold compared to that of naked mNPs. Such plentiful uptake could be attributed to the multiple surface adhesion molecules (such as E-cadherin and epithelial cell adhesion molecules) on the membrane with the homotypic tumor self-recognition ability.^39^ For further verification, we comparatively investigated the uptake of CmNPs (labeled with Cy5, Fig. S15†) into different cells, including 4T1, CT26, J774A.1, and HUVEC. As expected, a large number of CmNPs with marked fluorescence were observed in 4T1 cells rather than other cells (Fig. 3b). Once the mNPs were cloaked with different CMs sourced from the 4T1, CT26, J774A.1 or HUVEC cells, 4T1 cells exhibited much higher uptake of CmNPs compared to their counterparts cloaked with other CMs (Fig. S16†), again confirming the homotypic targeting performance of cloaked CmNPs.

To evaluate the targeting capacity *in vivo*, we next intravenously injected the formulations into the 4T1-xenografted mice and determined the intratumoral accumulation *via* time-elapsed PA signals (Fig. 3c and S17†). Although the mNP and CmNP groups shared similar kinetics in the tumor area, a much brighter PA signal was observed in the CmNP group, and the value of the CmNP group was 3–4 times higher than that of the mNP group. This effect should be attributed to the improved stability and targeting capacity sourced from the CM camouflage. For further verification, we simultaneously inoculated the mice with two different types of tumor xenografts at parallel sites (CT26 tumor on the left and 4T1 tumor on the right) and utilized *in vivo* fluorescence imaging to investigate the biodistribution of the intravenously injected formulations (labeled with Cy7). As shown in Fig. 3d, the accumulation of mNPs and CmNPs in both 4T1 and CT26 tumors again shared similar kinetics with the peak at 24 h. However, CmNPs showed much superior accumulation compared to that of mNPs in the 4T1 tumor area, while their signals in the CT26 tumor area were inconspicuous (Fig. 3e). In this case, the indicator ratio (I_4T1/CT26_) for recognition specificity in the CmNP group reached up to 5, which was markedly higher than the value of the mNP group. Such a disparity could also be observed in the excised tissues and histological sections (Fig. S18 and S19†), again demonstrating the specific targeting recognition ability to homologous tumors sourced from CM camouflage.

### Phototherapy performance of CmNPs *in vitro*

**Fig. 4:**
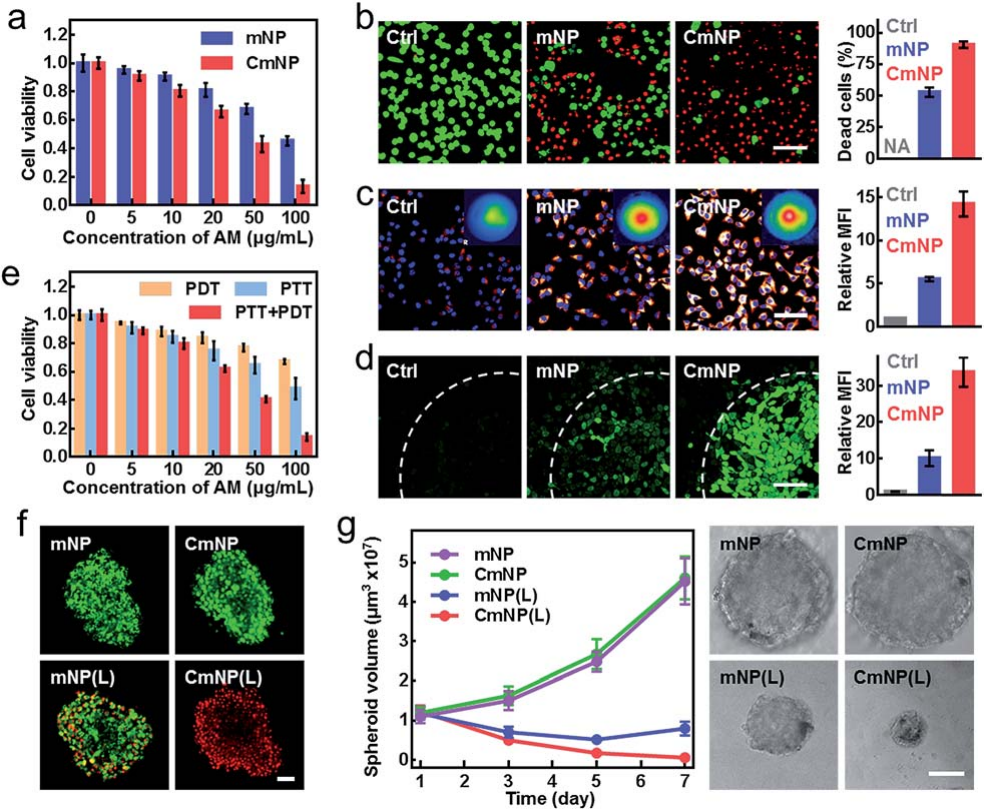
*In vitro* phototherapy. (a) Cell viabilities of 4T1 cells after treatment with different concentrations of mNPs or CmNPs, followed by irradiation. (b) Live/Dead staining of 4T1 cells after being treated with 100 μg mL^–1^ of mNPs or CmNPs, followed by irradiation (scale bar: 50 μm, green: calcein-AM stained live cells, red: PI stained apoptotic cells). (c) Immunofluorescence analysis of 4T1 cells labeled with HSP90 antibody after NIR laser irradiation (inset: thermal images, scale bar: 50 μm, glow: HSP90, blue: nuclei). (d) Intracellular ^1^O_2_ generation after irradiation examined by CLSM (scale bar: 50 μm, 808 nm, 1 W cm^–2^, 8 min. The cells were partly covered with a light spot showing the boundary). (e) Quantitative viability detection of 4T1 cells after PDT, PTT or simultaneous PDT/PTT treatment. (f) Live/Dead staining of 4T1 MCTS at 24 h after different treatments (scale bar: 50 μm). (g) Time-dependent change of the MCTS volumes and representative photos of the MCTS on day 7 after the treatments (scale bar: 250 μm). All data represent the means ± s.d. (*n* = 3).

The excellent targeting properties of CmNPs encouraged us to explore their phototherapy performance. As shown in Fig. 4a, the viability of 4T1 cells after 808 nm irradiation exhibited a dose-dependent effect. Owing to the improved stability and targeting capacity, the value in the CmNP group kept lower than that in the mNP group. The superior phototherapy performance of CmNPs was further confirmed by using calcein-AM and propidium iodide (PI) as probes to indicate the live and dead 4T1 cells, respectively (Fig. 4b), wherein treatment with CmNPs resulted in more dead cells (indicated by the red color). To gain a deeper insight, we also investigated the dual functions of PDT and PTT at the concentration of 100 μg mL^–1^. After irradiation, a high level of local hyperthermia occurred in the CmNP group, while the temperature in the PBS group was almost unchanged (Fig. 4c inset). Accordingly, we observed the highest expression of HSP90 in CmNP-treated cells, indicating a superior PTT for hyperthermia treatment (Fig. 4c). With respect to PDT, irradiation of the PBS group exhibited little ^1^O_2_ (detected by DCFH-DA with a green signal). Notably, CmNPs again outperformed mNPs, since the ^1^O_2_ generation in the CmNP group was more than 3-fold of that in the mNP group (Fig. 4d). Once the hyperthermia from PTT was cooled by an ice-bath or the ^1^O_2_ from PDT was absorbed by quenching, the cell viability was significantly recovered, in turn demonstrating the orchestration of PDT and PTT for potent cytotoxicity (Fig. 4e and S20†).

In addition to the 2D culture system, we also evaluated the phototherapy performance on a 3D multicellular tumor spheroid (MCTS) model, which could closely mimic the native structure of tumor tissue and therefore provided a more accurate antitumor estimation.^40^ As shown by the Live/Dead assay in Fig. 4f, all cells in MCTS remained alive (represented by a green color) in both the mNP and CmNP groups without irradiation, again suggesting good biocompatibility. When irradiation was added, a few dead cells appeared in the mNP group, while almost all cells in the CmNP group were dead. Such a disparity further resulted in different effects on MCTS growth (Fig. 4g). As expected, the non-irradiation group showed little effect on MCTS development. Upon irradiation, the MCTS volumes decreased in both mNP(L) and CmNP(L) groups. Comparatively, the MCTS in the CmNP(L) group shrank to a much smaller level, again confirming a more potent phototherapy effect.

### *In vivo* antitumor efficacy of CmNPs

**Fig. 5:**
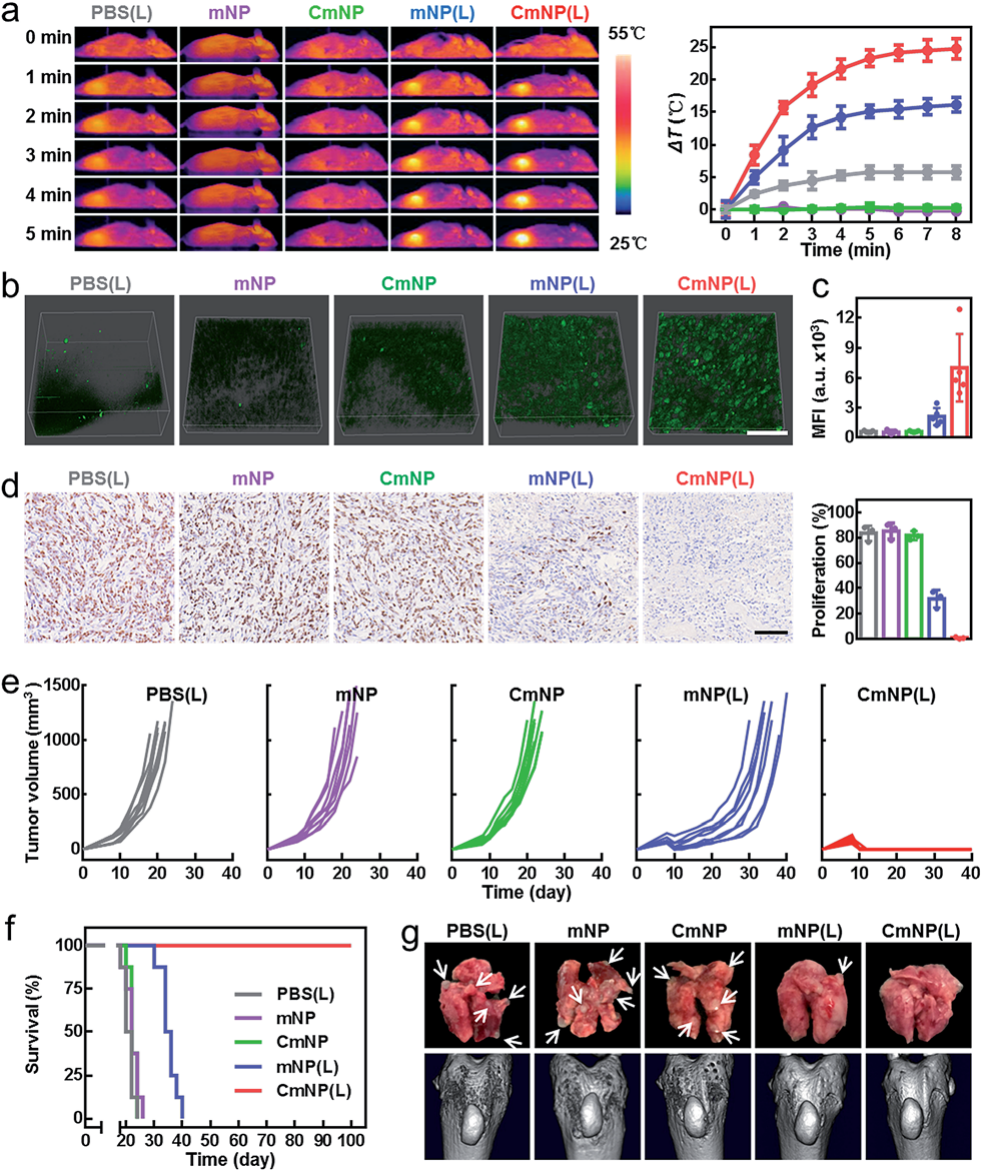
*In vivo* simultaneous PDT/PTT therapy. (a) Representative NIR thermal imaging and time-dependent temperature increase in 4T1 tumors irradiated by an 808 nm laser at 24 h after intravenous injection (1 W cm^–2^, 8 min). All data represent the means ± s.d. (*n* = 3). (b) Two-photon fluorescence imaging of DCFH-DA in tumors after different treatments (scale bar: 50 μm). (c) Mean fluorescence intensity of DCFH-DA in tumors analyzed by flow cytometry. All data represent the means ± s.d. (*n* = 5). (d) Ki67 of tumor sections collected at 12 h after irradiation (scale bar: 50 μm). (e) Tumor growth curves of different treatment groups over a 40 day period (*n* = 8). (f) Survival rates of 4T1 tumor-bearing mice (*n* = 8). (g) Representative photos of lung tissues and computed tomography (CT) imaging of spontaneous tibia metastasis collected from different treatment groups (the white arrows indicate metastatic nodules).

Encouraged by the above results, we finally evaluated *in vivo* therapeutic efficacy. To this end, 4T1 tumor-bearing mice were randomly divided into five groups: PBS(L), mNP, CmNP, mNP(L), and CmNP(L). *Via* the hyperthermia effect, we first imaged the infrared thermographic maps and monitored real-time temperature changes in the tumor area (Fig. 5a). Compared to the stable temperature in the non-irradiation group, the temperature in the mNP(L) group gradually increased to 48.3 °C, while it climbed up to 57.1 °C in the CmNP(L) group. To estimate the ^1^O_2_ production in tumors, DCFH-DA was intratumorally administered for *in situ* imaging by multiphoton laser confocal scanning microscopy and subsequent quantitative assay by flow cytometry (Fig. 5b and c). In the non-irradiation groups, little ^1^O_2_ signaling was found either *in situ* or *ex vivo*. Once upon irradiation, a much brighter signal could be observed in the CmNP(L) group, and the intensity was 3.3 times greater than that in the mNP(L) group.

The different photothermal effects and ^1^O_2_ generation cooperatively resulted in distinguishing the inhibition of tumor cell proliferation (detected by the expression of the Ki67 indicator in Fig. 5d). As could be seen, the mNP(L) group and CmNP(L) group showed moderate and significant inhibition of Ki67 expression, respectively. Correspondingly, mNP(L) treatment slightly suppressed tumor development, and the mice gradually died within 40 days. In contrast, the tumor could be completely eliminated in the CmNP(L) group, and the survival rate was 100% during 100 day observation (Fig. 5e and f, S21†). As metastasis to distant lung and bone tissues was always observed for breast cancer in the clinic, the anti-metastasis performance was also evaluated (Fig. 5g, S22†). Compared with other groups that showed metastatic foci in the lungs and metastasis-induced erosion in the tibia to different extents, no signs of metastasis were observed in the CmNP(L) group, again confirming the superior phototherapy performance of CmNPs. Considering the safety concerns of antimony,^41^–^43^ the biosafety was systematically evaluated again. Few abnormalities were found in all markers in CmNP(L) treated mice (Fig. S23, Table S1†). Such a good safety should be attributed to the superior targeting capacity of CmNPs, which could decrease the exposure of antimony in normal organs. Therefore, the safety concerns of CmNPs could be eliminated. All results indicated that the developed CmNPs held great promise for cancer theranostics.

## Conclusions

In summary, we succeeded in significantly improving the stability of mNPs by the cooperation of dimension optimization, size control, and CM camouflage, which meanwhile endowed the mNPs with homotypic targeting capacity at tumor sites. The resulting CmNPs thus exhibited not only satisfactory PA/photothermal multimodal imaging but also potent PDT/PTT combination anticancer therapy with no notable side effects. The maximized theranostic performance *in vivo* therefore supported CmNPs as a safe and high-performance modality to fight against cancer.

## Animals and Tumor Models

Four-week-old Balb/c mice were purchased from Vital River Laboratories (Beijing, China). Animals received care in accordance with the Guidance Suggestions for the Care and Use of Laboratory Animals. The animal protocol was approved by the Institutional Animal Care and Use Committees at the Institute of Process Engineering, Chinese Academy of Sciences. A transplantable breast cancer model was established by subcutaneously inoculating 4T1 cells (5 × 10^5^ cells) into the flank of Balb/c mice. The tumor volume was calculated as (tumor length) × (tumor width)2/2.

## Author contributions

H. Y. Xie and W. Wei conceived and designed the study; G. Lu, C. Lv, W. Bao, F. Li, F. Zhang, L. Zhang, S. Wang, X. Gao and D. Zhao performed the research and experiments; G. Lu, H. Y. Xie and W. Wei wrote and revised the manuscript. All the authors revised the manuscript and approved the submission.

## Conflicts of interest

There are no conflicts to declare.

## Supplementary Material

Supplementary information
