# Supplementary material for: Antimonene with two-orders-of-magnitude improved stability for high-performance cancer theranostics
Source: Chem Sci. 2019 Apr 2;10(18):4847–53. doi: 10.1039/c9sc00324j (PMC6520929; doi:10.1039/c9sc00324j)
Supplement: Supplementary file 1 [file SC-010-C9SC00324J-s001.pdf]

Electronic Supplementary Material (ESI) for Chemical Science.
This journal is © The Royal Society of Chemistry 2019

Antimonene with Two-Orders-of-Magnitude Improved Stability for High-Performance Cancer Theranostics

Guihong Lu,[a] Chengliang Lv,[a] Weier Bao,[b] Feng Li,[a] Fan Zhang,[a] Lijun Zhang,[b] Shuang Wang,[b] Xiaoyong Gao,[b] Dongxu Zhao,[a] Wei Wei*,[b] and Hai-yan Xie*,[a]

a School of Life Science, Beijing Institute of Technology, No.5 South Zhong Guan Cun Street, Beijing 100081, China
b State Key Laboratory of Biochemical Engineering, Institute of Process Engineering, Chinese Academy of Sciences, 1 North 2nd Street, Zhong Guan Cun, Beijing 100190, China

Experimental Section

Materials and Reagents. Antimony (Sb) powder was purchased from Sigma-Aldrich. N-methyl-2-pyrrolidone (NMP) was purchased from J&K Scientific Ltd. RPMI 1640 medium, Dulbecco's modified Eagle's medium (DMEM) and fetal bovine serum (FBS) were obtained from Gibco Life Technologies (AG, Switzerland). Trypsin-EDTA, penicillin-streptomycin and Hoechst 33342 were purchased from Life Technologies. Calcein-AM and propidium iodide (PI) were obtained from Invitrogen (USA). 2',7'-dichlorofluorescin diacetate (DCFH-DA) was purchased from Solarbio® life sciences. Hepes B buffer (2.38 $g{\cdot}L^{-1}$ Hepes, 0.476 $g{\cdot}L^{-1}$ $MgCl_2$, 0.292 $g{\cdot}L^{-1}$ EDTA, 0.154 $g{\cdot}L^{-1}$ DTT, 0.746 $g{\cdot}L^{-1}$ KCl, pH = 7.6) and Hepes C buffer (11.914 $g{\cdot}L^{-1}$ Hepes, 5.844 $g{\cdot}L^{-1}$ NaCl, 13.492 $g{\cdot}L^{-1}$ KCl, pH = 7.6) were prepared as described previously.[1]

Cell Culture. The 4T1 murine breast tumor cells were cultured in RPMI 1640. J774A.1, HUVEC and CT26 cells were cultured in DMEM, and all cell media were supplemented with 10% FBS and 1% (v/v) penicillin-streptomycin. The cells were incubated in a 5% CO_2 incubator at 37 °C. Red blood cells were obtained from mice.

Preparation of AM NPs and AM NSs. Different-sized AM NPs were prepared by a modified one-pot liquid exfoliation of bulk antimony (Sb) with a distinct probe followed by differential centrifugation. IIn detail, the Sb powder (50 mg/mL) was dispersed in 2 mL NMP and then sonicated using ultrasonic cell disruption system (JY92-IIN, Ningbo Scientz Biotechnology Co.,LTD.) for 8 h with an Φ6 ultrasound probe (1 000 W, On/Off cycle: 2 s/4 s), followed by an ultrasonic cleaning system (SB-5200DTDN, Ningbo Scientz Biotechnology Co.,LTD.) for 3 h (300 W). Both of the two sonication processes were in ice-bath. Then, the resulting solution was centrifuged to discard the unexfoliated bulk antimony (3 000 rpm × 10 min), and the lNP, mNP and sNP were obtained by centrifuging at 5 000 rpm, 5 000-10 000 rpm and 10 000-30 000 rpm, respectively. Specifically, the head of ultrasound probe were designed like jags (figures as follow). Specifically, the head of ultrasound probe were designed like jags. During 'on cycle', the irregular sound wave from the jag like head made AM move very rapidly and collide with the jags in all directions. The fast movement and incessant collision made the antimony turn around between the jags with nanoparticles obtained instead of sheets. The AM NSs were prepared with a normal ultrasound probe at a low-power ultrasound (300 W) according to a reported method.[2] All AM were finally resuspended in NMP and washed with water before use.

Preparation of the Cell Membrane Fragments. The cell membrane (CM) fragments were prepared as described previously.[3] Briefly, the 4T1 cells were suspended in 2 mL Hepes B buffer with 1% EDTA-free protease inhibitor and disrupted using an IKAT18 basic homogenizer (IKA, Germany) at 4 °C for 1 h. Then the homogenized solution was centrifuged at 10 000 g for 10 min, and the collected supernatants were laid on a discontinuous sucrose density gradient consisting of 30% (w/v), 40% (w/v) and 55% (w/v) sucrose. After ultracentrifugation at 28 000 rpm for 2 h, the 30% band was collected and ultracentrifuged at 28 000 rpm for 30 min to obtain the membrane

fragments, which were stored in Hepes C buffer at -20 °C. Other kinds of cell membrane fragments were obtained in a manner similar to that used for 4T1 cells. The concentrations of the cancer cell membranes were quantified by BCA assay.

Modifying AM with Cell Membrane Fragments. Firstly, the collected CM fragments were extruded through a 200 nm porous membrane to form CM vesicles. After that, the mNP was added (1:3 in mass), and then they were coextruded through a 100 nm porous membrane. The mechanical force and membrane fluidity cooperated to facilitate the mNP to cross the lipid bilayers, resulting in vesicle-particle fusion. The extrusion process was repeated for 10 times to prepare CmNP with uniform size, continuous and complete membrane coating. Finally, the production was centrifuged at 10 000 g to remove excessive CM.

To verify the cell membranes on the mNP particles by fluorescence imaging, the CmNP was fixed on glass bottom dishes at 37 °C for 1 h and then washed with PBS, followed by incubation with 1'-dioctadecyl-3,3,3',3'-tetramethylindodicarbocyanine (DiD) for 1 h. Then, the nanoparticles were viewed by confocal laser scanning microscopy (CLSM, UltraVIEW VoX, PerkinElmer, USA).

Characterization of NPs and NSs. The samples were prepared by dropping a diluted AM NSs and NPs onto mica film, and keeping for 1 h to obtain a dry surface. AFM imaging was carried out using a Bruker-Fastscanbio (Tip Model: Fastscan-C, Material: Silicon Tip on Nitride Lever). For TEM sample preparation, the uncoated NPs dispersed in ethanol or CmNP dispersed in PBS was dropped onto 200 mesh C-coated Cu grid, and then the TEM images were recorded on HT-7700 (HITACHI, Japan) operating at an accelerating voltage of 120 kV. The hydrodynamic diameter and zeta potential of the NPs was detected by a Malvern Zeta Sizer (Nano ZS). Raman spectra (LabRAM HR800, Horiba Jobin Yvon, France) and X-ray diffraction (XRD, Empyrean, PANalytical B.V., Holland) were used to analyze the crystal structure of the NPs. The UV-Vis-NIR spectrum was obtained with a U-3900 spectrophotometer (HITACHI). Electron spin resonance (ESR) (JEOL-FA200, Japan) was performed using a Bruker EMX plus model spectrometer operating with an X-band frequency of 9.4 GHz at room temperature. The concentration of AM was determined by ICP-MS (iCAP Qc, Thermo Scientific, USA). To examine the stability of the CmNP, the particles were dispersed in PBS or FBS (10%) and stored at 4 °C for one week. The diameter and zeta potential (10 μL sample to 1 mL water) of the particles was measured every day.

***In Vitro* Stability of NPs and NSs.** To compare the stability of the different forms of AM (sNP, mNP, lNP and NS), AM-based particles were individually dispersed in water for 10 min, followed by centrifugation at 20 000 g for 30 min. The degraded antimony oxides in the supernatant were determined by ICP-AES. Otherwise, different forms of AM with the same concentration (300 μg/mL) were maintained in a horizontal shaker at 37 °C, and then the absorption at 808 nm was detected at predetermined time intervals.

To future comparatively investigate the stability of sNP, mNP and CmNP, the NPs were individually dispersed in PBS with the same concentration (100 μg/mL) and maintained in a horizontal shaker at 37 °C. At predetermined time intervals, one aliquot of each solution was centrifuged at 20 000 g for 30 min. The degraded antimony oxides in the supernatant were determined by ICP-AES. Otherwise, the mNP and CmNP with the same concentration were maintained in a horizontal shaker at 37 °C, and the absorption spectra and photothermal heating curves were detected at predetermined time intervals (808 nm laser, 1 W cm^{-2}, 8 min).

Photothermal Performance of AM. 500 μL of NSs and NPs solutions of different concentrations (0-300 μg/mL) were added into an Eppendorf tube and irradiated for 8 min (808 nm, 0.8 W/cm^2). The infrared thermographic maps and temperature change of the aqueous solution were recorded using an infrared thermal imaging camera (FLIR E40).

Detection of $^1O_2$ Generation in Solution. To verify the involvement of $^1O_2$ in the fluorescence decay of ABDA, CmNP was suspended in 1 mL water containing 10 μM ABDA dye. As a control, 1 mM sodium azide was added along with CmNP. The solution was placed in a cuvette and irradiated with an 808 nm laser (0.8 W/cm^2), and the fluorescence of ABDA (upon excitation at 380 nm) was measured by a Fluoromax-4 spectrofluorometer (HORIBA JOBIN YVON). To verify the involvement of $^1O_2$ by DPBF, 40 μL DPBF (1 mg/mL) was added into 1 mL CmNP, and the solution was kept in the dark for 30 min. After irradiation with an 808 nm laser (0.5 W/cm^2), the UV-vis spectra of the samples were measured at different time intervals with a U-3900 spectrophotometer.

***In Vitro* PA Imaging.** The PA signals of CmNP with different concentrations were measured using a real-time multispectral optoacoustic tomographic imaging system (MOST inVision 128, iThera, Germany). The signal intensity changes were calculated by analyzing the interest ROI of the images.

***In Vitro* NIR-induced Degradation.** The mNP or CmNP were individually dispersed in PBS (200 μg/mL) and exposed to an 808 nm NIR laser for 5 min (0.8 W/cm^2), followed by natural cooling to room temperature. The irradiation and cooling were repeated 3 times. The photothermal heating-cooling curves and the absorption spectra after every cycle were evaluated.

***In Vitro* Toxicity Evaluation.** The 4T1 cells were seeded into 96-well plates at a density of 5×10^3 cells per well and incubated for 24 h. Then, different concentrations of mNP or CmNP (0-100 μg/mL) were added to the cells and incubated for another 6 h. Cell viability was determined using a standard CCK-8 assay (Beyotime, China).

***In Vivo* Toxicity Evaluation.** The *in vivo* toxicity of AM was preliminarily evaluated by serum biochemistry and H&E stained histology. In brief, CmNP (3 mg kg^{-1}) was injected (i.v.) into Balb/c mice. Blood was collected for serum biochemistry before injection and at different time points post injection (0, 1, 3, 7, 14, 30, 60, 90 days).

The body weight change in the mice was also recorded at different time points. At the end of the experiment, the major organs were collected at day 100 and sectioned into 10 μm slices, stained with H&E and examined by an automatic multispectral imaging system.

***In Vitro* Homologous Targeting capability of CmNP.** The 4T1 cells were seeded into 24-well plates and incubated for 24 h. Then the cells were treated with different concentrations (0-100 μg/mL) of mNP or CmNP for 1 h. The cells were washed with PBS, and then the particles bound to cells were determined by ICP-AES.

To investigate the uptake of NPs by different cells, 4T1 cells, HUVEC, J774A.1 or CT26 cells were seeded into glass bottom dishes and incubated for 24 h and then treated with mNP or CmNP (20 μg/mL) for 1 h. For microscopy imaging, the nuclei were stained with Hoechst 33342 and imaged by CLSM. Hoechst 33342 was excited with UV, emitting 450-500 nm fluorescence. The mNP were excited using a 633 nm laser, and emission was collected in the range of 628-638 nm. For flow cytometry analysis, the mNP were modified with DSPE-PEG (Cy5) before being coated with cell membrane. The collected cells were analyzed by flow cytometry (BECKMAN COULTER, Cytoflex LX).

To study the uptake of different membrane modified mNP by 4T1 cells, 4T1 cells were seeded into glass bottom dishes and incubated with CT26, J774, HUVEC or RBC membrane modified mNP for 1 h and then analyzed by CLSM and flow cytometry.

Intracellular $^1O_2$ and Thermal Detection. For intracellular $^1O_2$ detection, 4T1 cells were incubated with 50 μg/mL mNP or CmNP for 1 h in dishes. Then, 10 mM DCFH-DA was added and incubated for 30 min. Afterwards, the cells were irradiated with an 808 nm laser (1 W/cm^2, 8 min) and imaged by CLSM.

The expression of HSP90 protein was used as a representative indicator for intracellular thermal detection. The 4T1 cells were incubated with 50 μg/mL mNP or CmNP for 1 h in dishes. After being irradiated with an 808 nm laser, an anti-HSP90 antibody was added and incubated for 4 h. Then, the cells were washed with PBS three times and labeled with a fluorescent secondary antibody, followed by imaging by CLSM.

***In Vitro* Phototherapy.** The 4T1 cells (1×10^4 cells per well) were seeded in 96-well plates and incubated with different concentrations (0-100 μg/mL) of mNP or CmNP for 1 h. After washing with PBS, the cells were immersed in 100 μL fresh culture medium, and then irradiated with an 808 nm laser (1 W/cm^2, 8 min). The standard CCK-8 assay was used to evaluate the cell viability after the cells were incubated for another 24 h. For Live/Dead detection, the cells were incubated for another 4 h after irradiation. Then the cells were stained with calcein-AM for visualization of the live cells and PI for visualization of the dead cells. The experimental procedures were performed in accordance with the manufacturer's instructions.

To compare the synergistic PDT/PTT effect of CmNP on 4T1 cells, different concentrations of CmNP were added to the cells and incubated for 1 h. After washing

with PBS, the cells were immersed in 100 μL fresh culture medium and then irradiated with an 808 nm laser (1 W/cm^2, 8 min). To verify the PDT effect, an ice bath was used during laser irradiation to avoid photothermal effects. To verify the PTT effect, the cells were treated with 1 mM NaN_3 to avoid singlet oxygen generation. The cell viability and Live/Dead assays were evaluated as above.

Furthermore, 4T1 three-dimensional (3D) MCTS were also constructed to verify the phototherapy effects of mNP and CmNP. Briefly, 4T1 cells (1×10^4 per well) were seeded into a 1.5% (w/v) agarose precoated 96-well plate and incubated for 8 days to form MCTS. Then the MCTS was incubated with mNP or CmNP (100 μg/mL) for 24 h, followed by 808 nm laser irradiation (1 W/cm^2, 12 min). For the Live/Dead analysis, the MCTS were stained with a Live/Dead kit for 1 h, and evaluated in the same way as used for the 2D-cultured cells. To verify the *in vitro* phototherapy efficacy, the MCTS was further incubated for another 7 days and the volume of each MCTS was recorded every day.

***In Vivo* PA Imaging.** The 4T1 tumor-bearing mice were randomly divided into two groups (3 mice per group) and injected (*i.v.*) with mNP or CmNP (100 μL, AM: 3 mg/kg). *In vivo* PA images were acquired before injection and at different time intervals after injection by using a multispectral optoacoustic tomography imaging system (MOST inVision 128, iThera, Germany).

***In Vivo* Fluorescence Imaging.** The mice simultaneously bearing two different types of tumor xenografts at parallel sites (4T1 tumor at right and CT26 tumor at left) were injected (i.v.) with Cy7-labeled mNP or CmNP (100 μL, AM: 3 mg/kg). Fluorescence imaging was taken at different time intervals using an *ex/in vivo* imaging system (FX Pro, Kodak, Japan) with a 790 nm excitation wavelength and an 830 nm filter to collect the signals. In the *ex-vivo* imaging experiments, the mice were sacrificed at 24 h postinjection. The tumor and major organs were collected and imaged immediately. For histological evaluation, dissected tumors and organs were sectioned into 10 μm slices, and the nuclei of tumor cells were stained with Hoechst 33342. The fluorescence images of all sections were acquired on a laser scanning confocal microscope (Leica TCS SP5).

***In Vivo* Thermal Imaging**. The 4T1 tumor-bearing mice were randomly divided into five groups (3 mice per group) and individually treated with PBS(L), mNP, CmNP, mNP(L) or CmNP(L) (100 μL, AM: 3 mg/kg). At 24 h postinjection, the tumors in groups 1, 4 and 5 were irradiated by an 808 nm laser (1 W/cm^2, 8 min). The temperature change and infrared thermographic maps of the tumors were obtained by using an infrared thermal imaging camera.

***In Vivo* $^1O_2$ Detection.** The 4T1 tumor-bearing mice were treated in the same way as that for *in vivo* thermal imaging, except that 10 μL (10 mM) DCFH-DA was intratumorally injected into the tumor before irradiation. The fluorescence of the DCFH-DA in tumors was imaged by a multiphoton laser confocal scanning

fluorescence microscope (FV1200MPE-M, Olympus, Japan) with an 800 nm excitation and analyzed by Imaris 8.1.2. Subsequently, the tumors were collected and triturated into a single cell suspension, and the DCFH-DA signal in tumor cells was analyzed by flow cytometry.

Apoptosis Detection. To detect the proliferation of tumor cells in tumor tissues, tumors were collected at 12 h postirradiation, sectioned into 10 μm slices and stained according to a Ki 67 kit. All slices were imaged by an automatic multispectral imaging system (PerkinElmer Vectra II, USA).

***In Vivo* Phototherapy.** The 4T1 tumor-bearing mice were randomly divided into five groups (8 per group) and treated in the same way as that for *in vivo* thermal imaging. After laser irradiation (808 nm laser, 1 W/cm^2, 8 min), the tumor volume and body weight change of each mouse were recorded every other day. At the end of the experiment, the lung and hind leg of each mouse were collected to examine the metastasis of the tumors. The tibia metastasis was imaged by computed tomography (CT).

Statistical Analysis: Statistical analysis of the data was performed using the Student's test. All results were expressed as mean ± standard error unless otherwise noted. $^{**}P < 0.05$, $^{***}P < 0.01$.

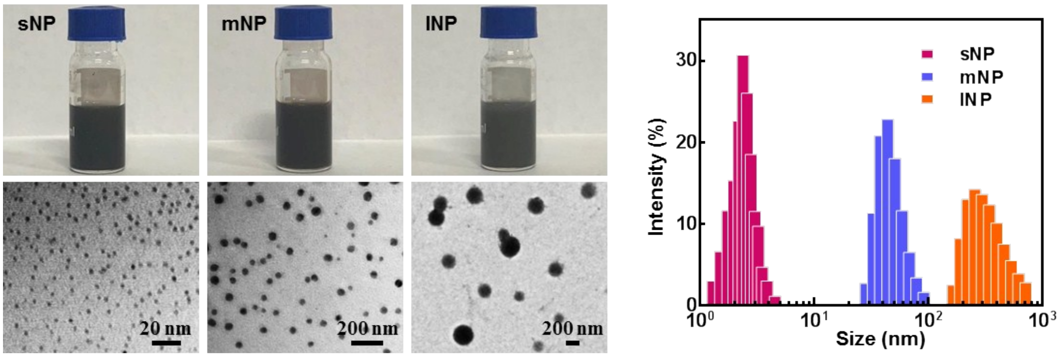

Figure S1. Photo image, TEM image and size distribution of NPs with different sizes.

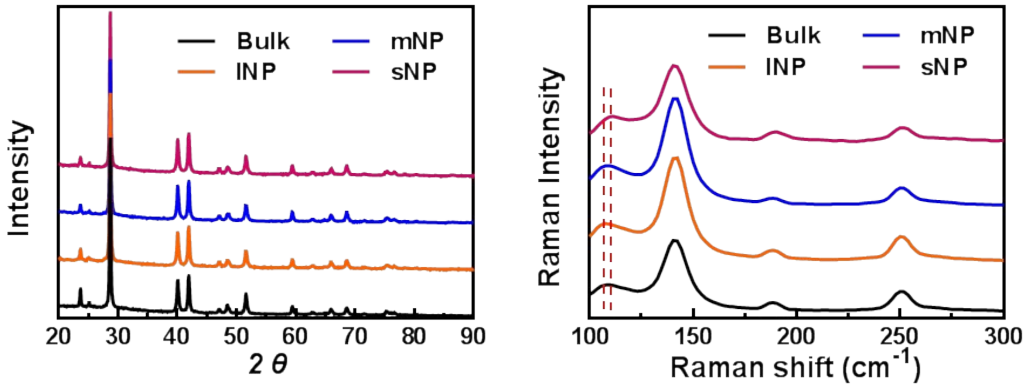

Figure S2. The XRD and Raman spectra of bulk antimony and NPs. The XRD spectra of different-sized NPs could be indexed as hexagonal Sb consistent with JCPDS No. 35-0732.[2] The NPs peaks in Raman spectra were all similar to bulk Sb (ca. 108 and 147 cm^{-1}),[4] although a slight shift of Raman spectra was observed compared with bulk antimony due to the sample thickness change.[5]

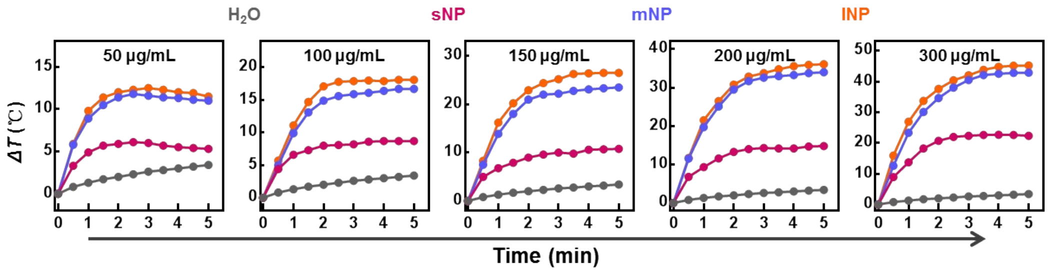

Figure S3. Time-dependent temperature changes of NPs during irradiation (808 nm, 0.8 W/cm^2). The increased temperature of mNP was much higher than that of sNP at the same concentration.

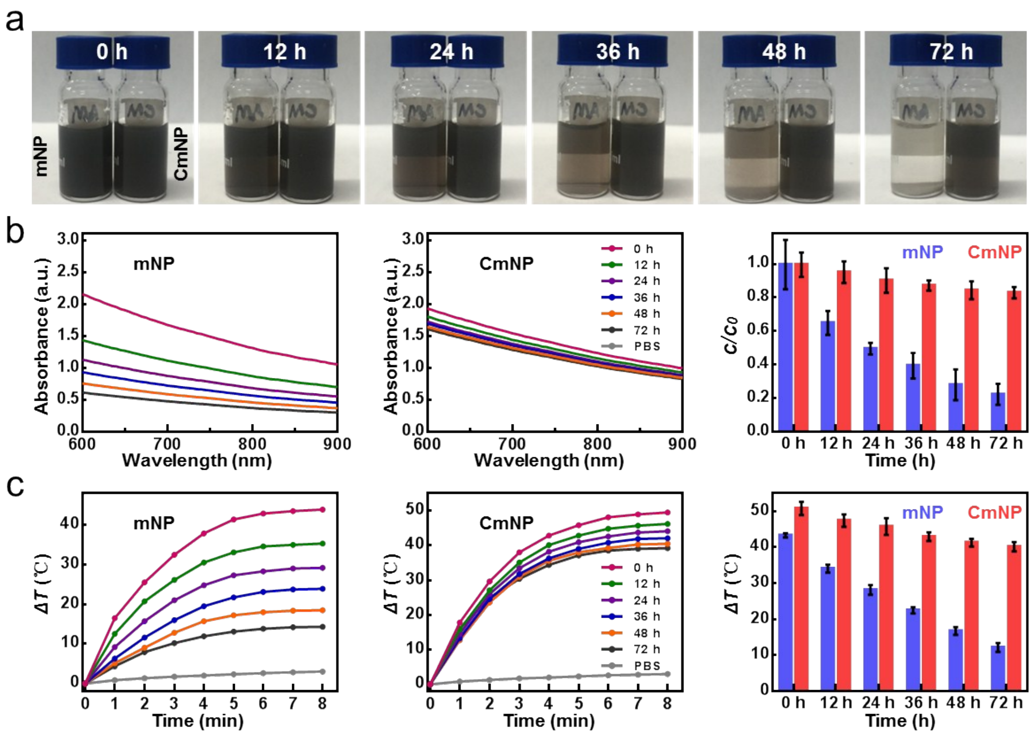

Figure S4. Stability evaluation of mNP and CmNP with the same amount of AM (300 μg/mL) in PBS. a) Photographs of the mNP and CmNP after being stored in PBS for different periods of time. b) The absorption spectra and variation of the absorption ratios (C/C_0) of the samples from a) at 808 nm. c) The photothermal heating curves and individual temperature change (ΔT) of the samples from a) (808 nm laser, 0.8 W/cm^2). CAM was more stable than AM, suggesting that CM coating could effectively protect AM from degradation.

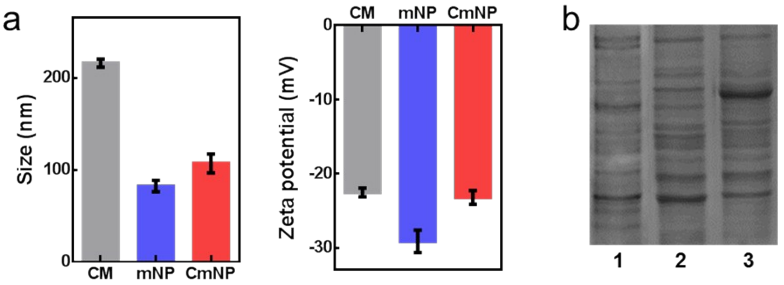

Figure S5. Cell membranes (CM) coating evaluation. a. Particle sizes and zeta potentials of CM, AM NPs and CmNP. The CmNP was approximately 20 nm larger than the pristine AM and possessed a similar surface zeta potential with that of native CM (-23.2 ± 0.9 mV). b. Sodium dodecyl sulfate polyacrylamide gel electrophoresis (SDS-PAGE) analysis of 1) whole 4T1 cells; 2) cell membrane of 4T1 cells; 3) CmNP. All results suggested that AM NP was successfully coated with CM.

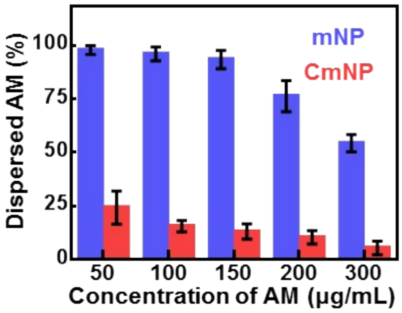

Figure S6. The dissolution mNP and CmNP at different concentrations after being dispersed in PBS for 24 h. All mNP were almost dispersed when the concentration was lower than 150 μg/mL. Furthermore, the dispersed NPs were more than 50% even at a high concentration of 300 μg/mL, while the CmNP exhibited a much improved stability.

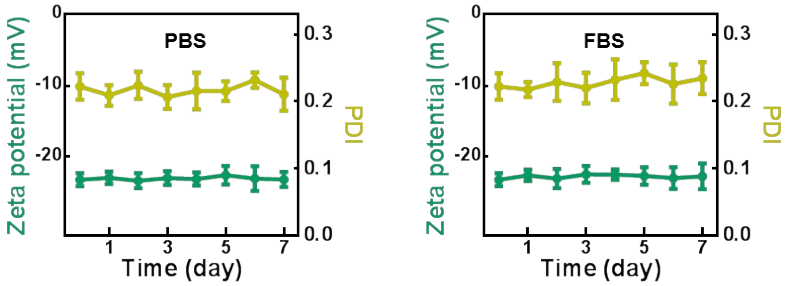

Figure S7. Zeta potential and polydispersity index (PDI) of CmNP in PBS or 10% FBS over one week. The Zeta potential and PDI remained relatively the same, which showed that the cell membrane covered the particle surface during the observation period, and also indicated the good stability of the CmNP.

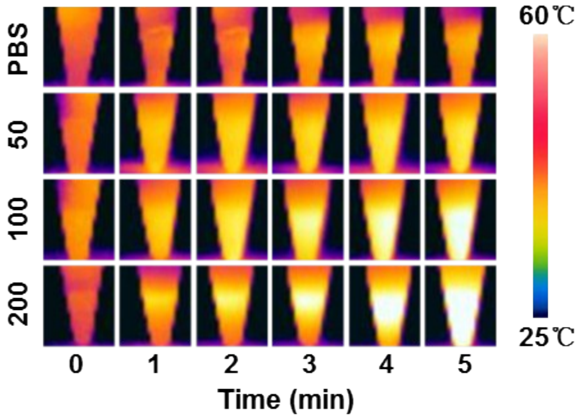

Figure S8. Thermal images of CmNP solution with different concentrations (0-200 μg/mL, 808 nm, 0.8 W/cm^2, 8 min).

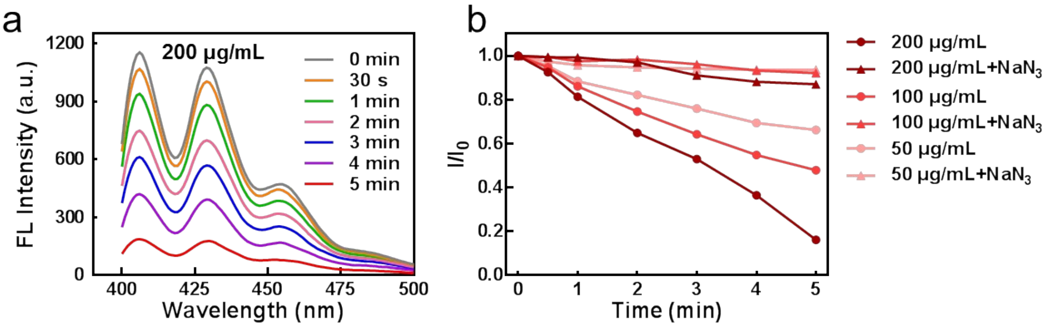

Figure S9. Verifying the $^1O_2$ generation from CmNP by ABDA. a) Time-dependent fluorescence decay of ABDA incubated with CmNP during irradiation (808 nm, 0.8 W/cm^2). b) Normalized fluorescence intensity of ABDA at 410 nm as a function of irradiation time. As a control, 1 mM sodium azide (a scavenger of $^1O_2$) was added along with CmNP. The fluorescence intensity of ABDA gradually decreased with irradiation time, illustrating the formation of endoperoxide. In addition, the decline rate of ABDA was proportional to the concentration of CmNP, but was almost prevented with sodium azide (NaN_3).

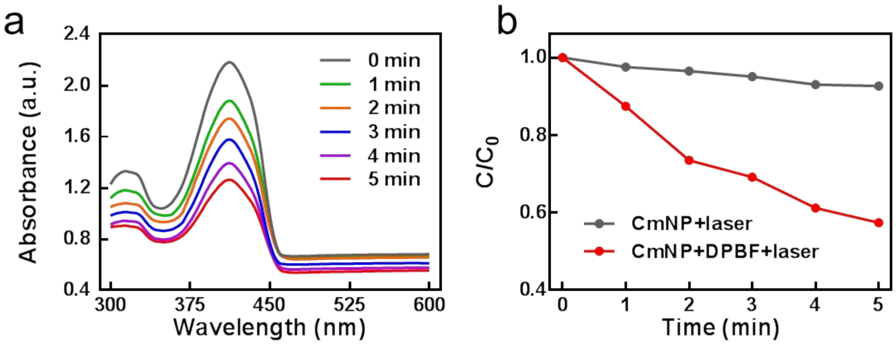

Figure S10. Verifying the $^1O_2$ generation from CmNP by DPBF. a) Time-dependent absorption spectra of DPBF incubated with CmNP during irradiation (AM: 200 μg/mL, 808 nm, 0.5 W/cm^2). b) Normalized absorbance of DPBF as a function of irradiation time. DPBF absorbance decay demonstrated the $^1O_2$ generation from AM. The results also demonstrated that CmNP could effectively generate $^1O_2$ under light irradiation, indicating its high feasibility for using as a PDT agent.

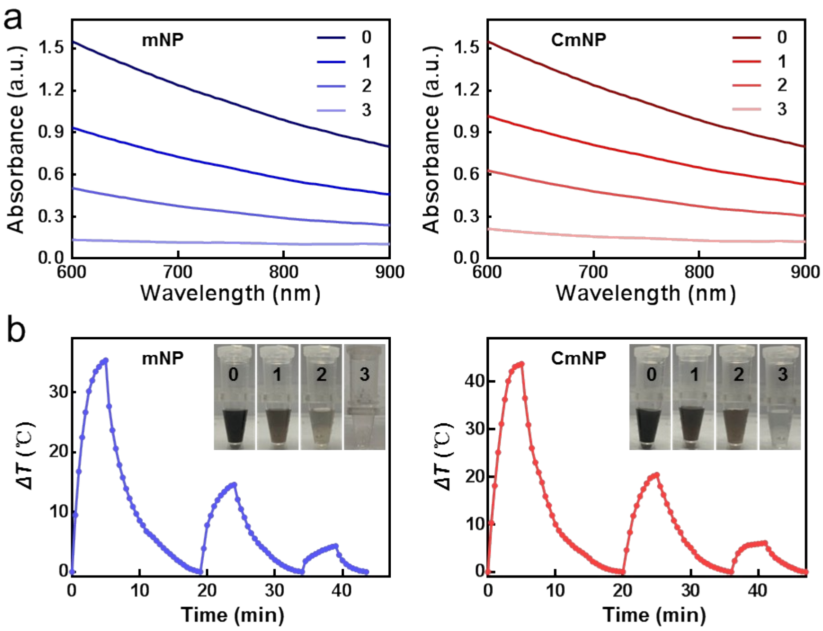

Figure S11. NIR-induced degradation of mNP and CmNP. The UV/Vis-NIR absorbance spectra a) and photothermal heating-cooling curves b) of mNP and CmNP with NIR laser irradiation. The degradation of CmNP was similar to that of mNP, indicating that the NIR-induced degradability of mNP was not influenced by the membrane coating.

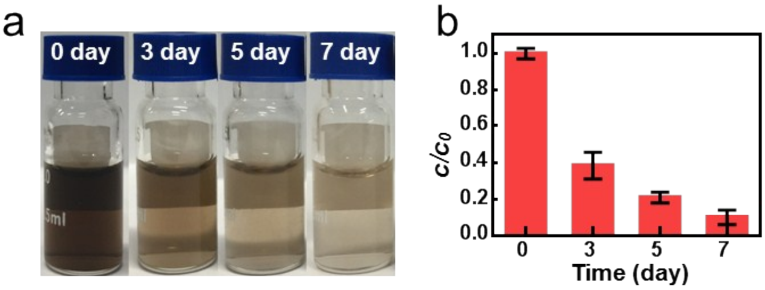

Figure S12. Degradation of CmNP at 37 °C (AM: 100 μg/mL). a) Photographs of CmNP after being stored in PBS for different periods of time. b) The variation of the absorption ratios (C/C_0) at 808 nm. The particles could be gradually dispersed at 37 °C even without NIR irradiation, thus enabled harmless clearance from the body in a reasonable period of time.

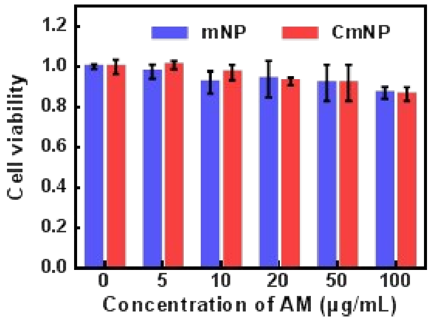

Figure S13. Cell viability after incubation with different concentrations of mNP or CmNP. No obvious cytotoxicity was observed. All data represent the means ± s.d. (n = 3).

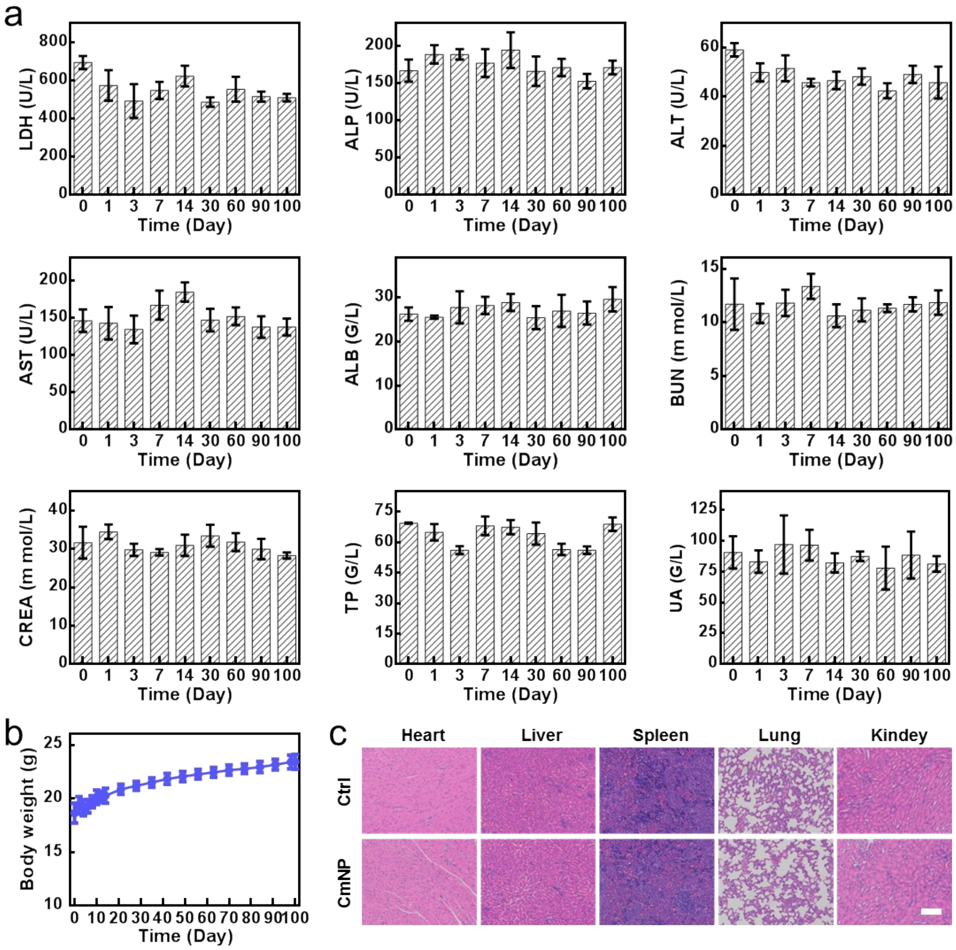

Figure S14. *In vivo* toxicity evaluation. a) Blood biochemical analysis, b) Body weight and c) Histological data (haematoxylin and eosin stained images) of CmNP treated mice. (Scale bar: 50 μm, Ctrl: health mice). According to the data, no weight and histological abnormalities in main organs were found, the blood biochemicals were also within normal ranges. These results demonstrated the good biocompatibility of the CmNP.

Abbreviations: LDH, lactate dehydrogenase; ALP, alkaline phosphatase; ALT, alanine aminotransferase; AST, aspartate aminotransferase; ALB, albumin; BUN, blood urea nitrogen; CREA, creatinine; TP, total protein; UA, uric acid.

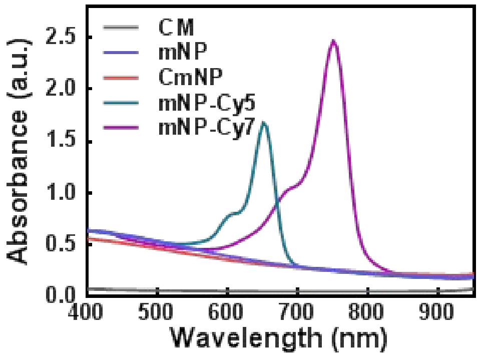

Figure S15. Absorbance spectra of CM and different formations of mNP. The absorbance properties of mNP were not influenced by CM coating, and mNP could be labeled with Cy5 or Cy7. To label mNP with fluorescence, 10 mg/mL DSPE-PEG (Cy5 or Cy7) was added to mNP solution in a 3:1 mass ratio at 37°C for 1 h. The excess dye molecules were removed by centrifugation and washed away with water. After which, some of the Cy5 or Cy7 labeled mNP were used to prepare CmNP.

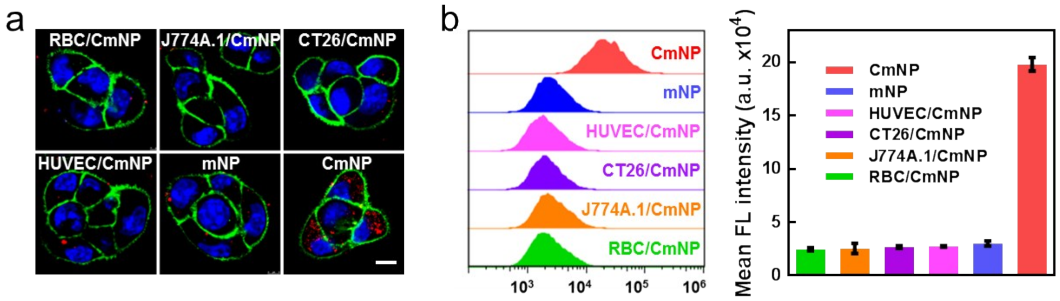

Figure S16. a) CLSM images and b) the corresponding flow cytometry analysis of 4T1 cells after incubation with different cell membrane modified mNP or bare mNP. (The concentration of AM in all cases was 20 μg/mL, scale bar: 5 μm, red: NPs, blue: nuclei, green: cell membrane). All data represent the means ± s.d. (n = 3).

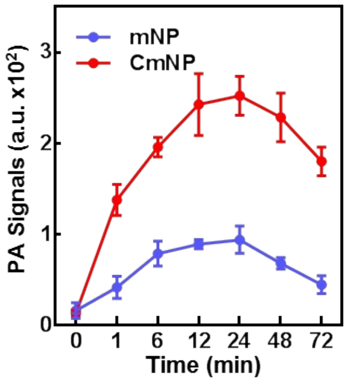

Figure S17. PA signal profiles of tumors at different time intervals from Figure 3c in manuscript. All data represent the means ± s.d. (n = 3).

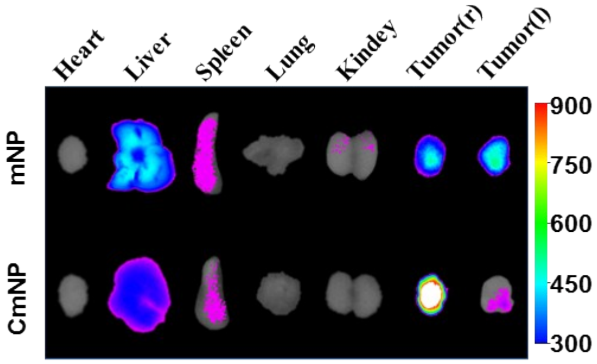

Figure S18. *Ex-vivo* fluorescence imaging of tumor and major organs of mice 24 h after injection. The results showed that most of CmNP accumulated in 4T1 tumor, while a majority of mNP distributed in other organs.

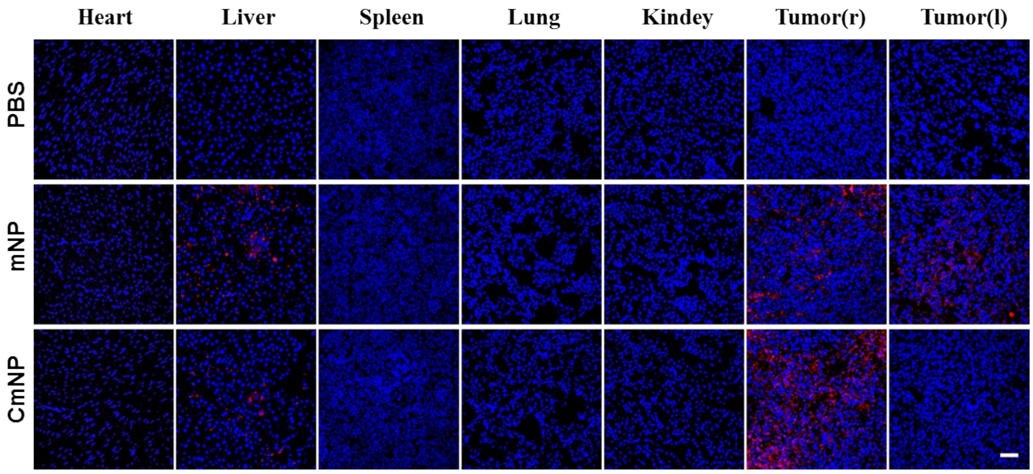

Figure S19. Histological section images of main organs and tumor with different treatments 24 h after intravenous injection (Scale bar: 50 μm, red: NPs, blue: nuclei). Consequently, much more CmNP could be observed in the sections of 4T1 tumor tissue.

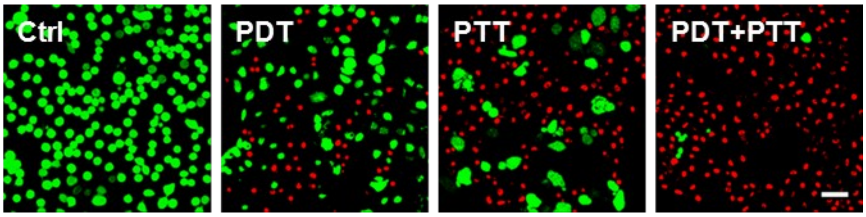

Figure S20. Confocal fluorescence images of 4T1 cells after PDT, PTT or simultaneous PDT/PTT treatment (Scale bar: 25 μm, viable cells were stained with green calcein-AM, dead/later apoptosis cells were stained with red PI; AM: 100 μg/mL; 808 nm laser, 1 W/cm^2, 8 min).

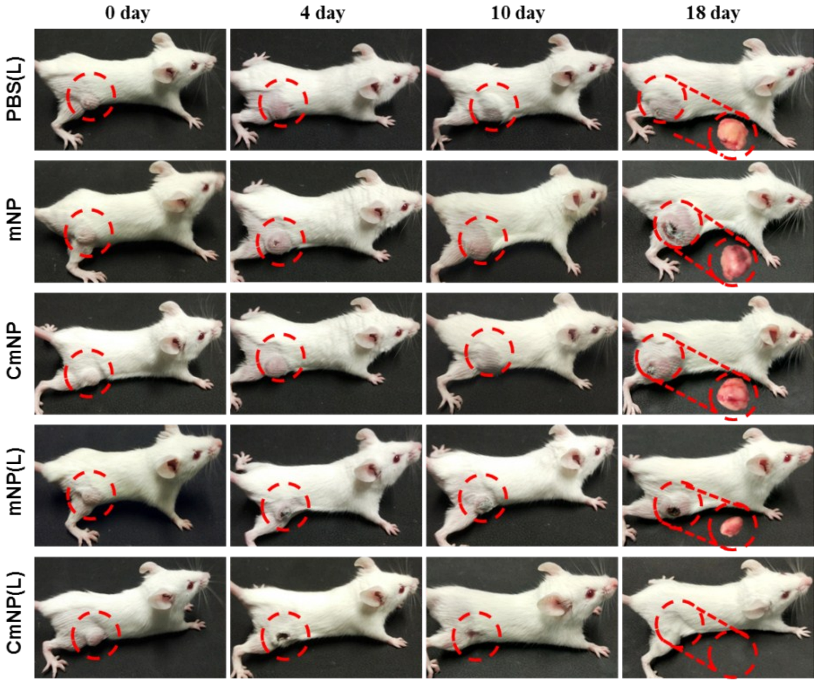

Figure S21. Representative photos of mice and the extracted tumors at the end of treatment.

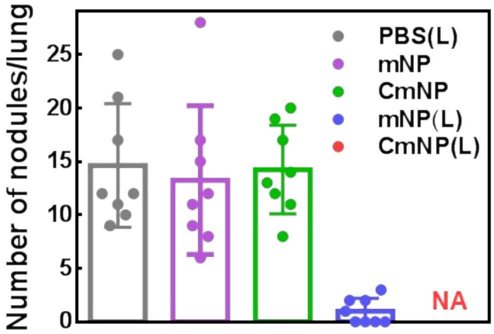

Figure S22. Quantitative analysis of pulmonary metastatic nodules for every group (n=8).

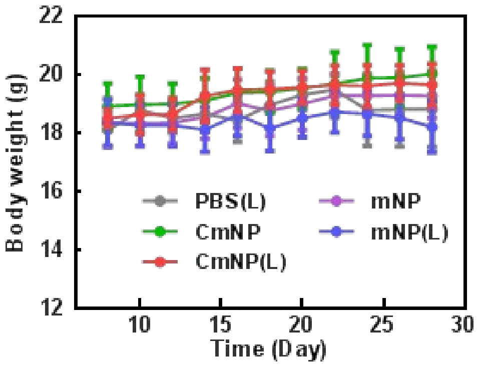

Figure S23. Body weight change curves of 4T1 tumor-bearing mice receiving different treatments. There were almost no changes in all groups. All data represent the means ± s.d. (n = 8).

Table S1. Blood biochemical analysis of mice after having received different treatments at 24 days. After phototherapy, the values of ALT, AST and LDH return to normal ranges, suggesting the superior therapy performance and the safety of CmNP. All data represent the means ± s.d. (n = 8).

	PBS(L)	mNP	CmNP	mNP(L)	CmNP(L)	Normal range
BUN (mmol/L)	12±2	11±1	12±2	11±3	12±1	8～33
ALT (U/L)	279±193	351±228	216±123	41±10	43±7	17～77
AST (U/L)	848±290	689±219	817±216	198±18	175±17	54～298
LDH (U/L)	3582±509	3978±565	4179±916	758±71	694±44	215～1024
ALP (U/L)	113±20	98±12	93±11	93±23	105±17	60～209

REFERENCES

1. Y. Lv, M. Liu, Y. Zhang, X. Wang, F. Zhang, F. Li, W. E. Bao, J. Wang, Y. Zhang and W. Wei, *ACS Nano*, 2018, **12**, 1350-1358.

2. T. Wei, J. Xiaoyuan, Z. Xianbing, L. Li, W. Junqing, Z. Ye, S. P. Er, L. Wenliang, K. Na and I. M. Ariful, *Adv. Mater.*, 2018, **30**, 1802061.

3. Z. Fan, L. Zhao, S. Wang, J. Yang, G. Lu, N. Luo, X. Gao, G. Ma, H. Y. Xie and W. Wei, *Adv. Funct. Mater.*, 2018, **28**, 1703326.

4. G. Pizzi, M. Gibertini, E. Dib, N. Marzari, G. Iannaccone and G. Fiori, *Nat. Commun.*, 2016, **7**, 12585.

5. J. Ji, X. Song, J. Liu, Z. Yan, C. Huo, S. Zhang, M. Su, L. Liao, W. Wang and Z. Ni, *Nat. Commun.*, 2016, **7**, 13352.
